# Which Ties Matter? Differential Effects of Family, Peer, and Community Support on Short-Video Engagement Among Older Adults

**DOI:** 10.3390/bs16040571

**Published:** 2026-04-10

**Authors:** Ziqing Yang, Xiaoxin Yu, Hao Gao

**Affiliations:** 1 School of Modern Circulation, Guangxi International Business Vocational College, Nanning 530007, China; 2530404020@st.gxu.edu.cn (Z.Y.); yiyu@163.com (X.Y.); 2 China-ASEAN School of Economics, Guangxi University, Nanning 530007, China; 3 School of Journalism and Communication, Nanjing Normal University, Nanjing 210097, China

**Keywords:** short-form video, social support, older adults, social empowerment, chained mediation, life satisfaction, aging, China

## Abstract

Short-form video (SFV) platforms have become a central part of older adults’ digital lives, yet their psychological implications remain theoretically contested. Drawing on social empowerment theory, Self-Determination Theory, attachment theory, and the displacement hypothesis, this study examined whether different sources of social support—family, peer, and community—exert differential effects on life satisfaction through SFV engagement and social connectedness. Survey data were collected from 385 community-dwelling Chinese older adults (mean age = 70.6 years) and analyzed using bootstrapped serial mediation models with 5000 resamples. The results revealed clear source differentiation, as family support most strongly predicted SFV engagement and showed the largest total association with life satisfaction, consistent with a social empowerment mechanism. Community participation showed a weaker but still positive association with engagement, whereas peer support was unrelated to engagement. Across pathways, higher SFV engagement was associated with lower social connectedness, while greater social connectedness was associated with higher life satisfaction. However, none of the chained indirect effects reached significance, suggesting that social support influenced life satisfaction primarily through direct rather than serially mediated pathways. These findings demonstrate the importance of disaggregating social support by source and contribute to a more precise framework for understanding older adults’ digital well-being.

## 1. Introduction

Global population aging represents one of the most significant demographic transformations of the twenty-first century, with profound implications for health systems, social policy, and everyday life. China stands at the forefront of this challenge, as by 2025, the population aged 60 and above had surpassed 323.38 million, accounting for 23% of the national population (National Bureau of Statistics of China, 2026). Yet this demographic shift is unfolding alongside—and increasingly intertwined with—a parallel transformation in digital technology adoption. According to the China Internet Network Information Center (CNNIC, 2025), internet users aged 60 and above have reached 161 million, with short-form video (SFV) platform penetration exceeding 60% among this cohort and average daily usage duration continuing to rise year on year. Platforms such as Douyin and Kuaishou have evolved from niche entertainment tools into primary infrastructures through which older adults seek information, maintain social connections, and structure leisure time—reshaping the psychological landscape of later life in ways that are only beginning to be understood.

This rapid adoption has attracted growing scholarly attention, yet findings remain deeply contested. A strand of research documents positive associations between SFV engagement and psychological well-being among older adults, noting benefits such as reduced loneliness, enhanced sense of social presence, and improved life satisfaction (R. Zhang et al., 2024; Nebenzahl-Elitzur et al., 2026). A contrasting body of work raises concerns about habitual or excessive use, linking high SFV engagement with social withdrawal, reduced offline participation, and potential high-intensity engagement patterns (Liu & Baumeister, 2016; Andreassen et al., 2017; Cui et al., 2026). This persistent tension signals not a methodological failure but a substantive one: aggregate assessments of use frequency obscure the social conditions that determine whether digital engagement promotes or undermines well-being. Understanding those conditions is the central ambition of the present study.

### 1.1. Competing Theoretical Accounts: Uses and Gratifications Versus Compensatory Internet Use

Two theoretical frameworks have dominated scholarly accounts of older adults’ digital media engagement, and their predictions point in opposite directions. Uses and Gratifications Theory (UGT; Katz et al., 1974) conceptualizes media users as active, purposive agents who select media to fulfill specific psychological and social needs—information seeking, entertainment, escapism, and social interaction chief among them. In older adults, UGT predicts that SFV engagement reflects deliberate, self-directed choices in the service of need fulfillment. When those needs are successfully met, engagement reinforces well-being rather than compromising it. This framing casts older adults not as passive victims of algorithmic design but as competent agents navigating digital environments on their own terms (Smock et al., 2011; Whiting & Williams, 2013).

Compensatory Internet Use Theory (CIUT; Kardefelt-Winther, 2014; LaRose et al., 2003), by contrast, argues that deficits in offline life fundamentally drive problematic or excessive online engagement. According to this view, older adults who lack adequate social support, meaningful daily structure, or satisfying interpersonal relationships turn to digital media as a substitute—a compensatory mechanism that temporarily alleviates distress but ultimately intensifies dependency and erodes real-world social capital (Caplan, 2010; Leung, 2007). CIUT thus predicts a negative relationship between offline social resources and digital media engagement: the richer one’s offline support, the less the drive toward online immersion.

For much of the past decade, these two frameworks were treated as mutually exclusive. Recent evidence, however, has begun to challenge this binary. Emerging evidence from older adult samples in China suggests that the relationship between offline social support and SFV engagement may be positive rather than negative. Results show that older adults with richer offline support appear to engage more intensively with digital media, not less, and this higher engagement is associated with, rather than detrimental to, psychological well-being (Cui et al., 2026). This empirical pattern directly inverts the CIUT prediction and sits uneasily within standard UGT accounts, which do not predict that the prior fulfillment of social needs would amplify media engagement. Existing theoretical frameworks, in other words, are insufficient to account for what the data consistently show.

We propose that this observed pattern reflects what we term a social empowerment mechanism: the process by which adequate offline social support—rather than suppressing the motivation for digital engagement—provides the psychological security that enables older adults to engage with SFV freely, autonomously, and without the anxious, compensatory quality characteristic of deficit-driven use. On this account, immersive SFV engagement among socially supported older adults is not a symptom of deprivation but an expression of confidence—a leisure choice made from a position of relational security rather than isolation. We use “social empowerment” as a descriptive label for this pattern and, in the section that follows, provide its theoretical foundation in Self-Determination Theory and attachment research. This theoretical elaboration constitutes one of the present study’s core contributions: moving from the observation that well-supported older adults engage more with SFV to an account of why this is so and under what conditions it holds.

### 1.2. Social Support, Psychological Security, and Digital Behavior: The Empowerment Mechanism

The social empowerment mechanism we have identified empirically can be given theoretical grounding through the combined resources of Self-Determination Theory (SDT; Deci & Ryan, 2000; Ryan & Deci, 2017) and attachment theory (Bowlby, 1969). SDT posits that human well-being depends on the fulfillment of three basic psychological needs: autonomy (the experience of volitional self-regulation), competence (the sense of effective action), and relatedness (the experience of meaningful connection with others). Crucially, SDT further proposes that the prior satisfaction of basic needs—particularly relatedness—creates the psychological conditions for autonomous, rather than controlled or compulsive, engagement with subsequent activities (Ryan & Deci, 2017; Przybylski et al., 2013). When older adults’ need for relatedness is durably met through offline social relationships, they approach digital leisure from a position of psychological security where they can engage deeply, step back freely, and experience immersion as chosen rather than driven. This is precisely the signature of empowered, rather than compensatory, digital behavior.

Attachment theory provides a complementary account at the relational level. Bowlby (1969) argued that secure attachment relationships function as both a “haven”—reducing anxiety when the individual is threatened—and a “secure base” from which confident exploration of the environment becomes possible. In the digital context, older adults whose relational needs are met by stable, secure offline bonds are free to explore digital environments—including immersive SFV engagement—without the existential anxiety that would accompany such behavior in conditions of relational deprivation. Social support, on this integrated account, does not merely permit digital engagement; it authorizes it as a legitimate, self-endorsed activity (cf. Przybylski & Weinstein, 2017). The empirical implication is straightforward: older adults with stronger family support—the source most closely approximating a secure attachment base—are expected to report the highest levels of SFV engagement (H1a).

**H1a:**  *Family support will positively predict SFV engagement, reflecting a relational security–based empowerment mechanism whereby secure family bonds enable autonomous digital leisure.*

**H1b:**  *Community participation will positively predict SFV engagement at a weaker magnitude than family support, reflecting a partial empowerment effect moderated by structural temporal constraints.*

**H1c:**  *Peer support will show a negligible or non-significant association with SFV engagement, reflecting the joint suppression of empowerment by social monitoring and activity displacement mechanisms.*

### 1.3. SFV Engagement, Social Connectedness, and the Displacement Risk

While social empowerment theory reframes the meaning of digital immersion, it does not rule out the possibility that sustained, high-intensity engagement may carry costs—particularly for social connectedness. The displacement hypothesis, first articulated in the context of television research (Kubey & Csikszentmihalyi, 1990) and subsequently applied to social media (Kraut et al., 1998; Verduyn et al., 2015), proposes that time-intensive media use competes with and displaces offline social interaction. For older adults, whose social networks are already subject to attrition due to bereavement, health decline, and residential mobility (Cornwell et al., 2008), this displacement risk may be particularly consequential.

Empirical research on SFV specifically provides some support for this concern. High-frequency SFV users report reduced engagement in face-to-face social activities (Cui et al., 2026), and longitudinal studies suggest that heavy passive social media consumption is associated with increases in loneliness over time (Twenge et al., 2020; K. Zhang et al., 2020). Among older adults, loneliness represents a major risk factor for poor health outcomes, cognitive decline, and reduced subjective well-being (Cacioppo & Hawkley, 2009; Victor & Bowling, 2012), making the potential erosion of social connectedness a serious concern even when initial engagement is autonomously motivated.

The consequences of diminished social connectedness for psychological well-being are well established. Loneliness—operationalized as the subjective experience of a discrepancy between desired and actual social connection—is consistently among the strongest predictors of reduced life satisfaction and poor mental health in later life (Peplau & Perlman, 1982; Russell, 1996; Holt-Lunstad et al., 2015). Conversely, maintaining a sense of social connectedness sustains the perception of being embedded in a network of meaningful relationships, which constitutes a core dimension of eudaimonic well-being (Ryff, 1989; Diener et al., 1985).

The displacement risk, however, is not expected to operate uniformly across support contexts. Older adults embedded in strong family relationships may buffer the connectedness cost of SFV engagement through the relational security that family ties provide; the displacement effect is therefore expected to be most pronounced where peer monitoring and social comparison are salient—that is, in the peer support pathway.

**H2:**  *SFV engagement will negatively predict social connectedness across all three pathways; this displacement effect will be most pronounced in the peer support pathway, where monitoring mechanisms heighten awareness of social withdrawal.*

**H3:**  *Social connectedness will positively predict life satisfaction, such that older adults with greater perceived social connectedness will report higher levels of subjective well-being, irrespective of support source.*

### 1.4. Beyond Total Support: The Case for Source Differentiation

The theoretical logic developed thus far—social support enabling empowered digital engagement, engagement potentially displacing social connectedness, diminished connectedness reducing satisfaction—captures the broad architecture of the process. However, a critical limitation of existing research is treating social support as a unitary construct. Most studies employ aggregate or composite measures that collapse distinctions between support from different sources (Lakey & Cohen, 2000; Thoits, 2011). This practice obscures theoretically and empirically significant heterogeneity.

House’s (1981) foundational typology distinguished social support not only by function (emotional, instrumental, informational, appraisal) but implicitly by source, recognizing that different relationships carry different normative structures and relational dynamics. In the context of older adults’ lives, the structural differences between family, peer, and community support are particularly pronounced—and may produce meaningfully different influences on digital behavior.

Family support is characterized by unconditional availability, continuity across the life course, and the absence of formal expectations of reciprocity (Antonucci & Akiyama, 1987). In Chinese cultural contexts, family relationships retain strong Confucian normative underpinnings that emphasize filial piety and mutual obligation (Silverstein et al., 2006; Cong & Silverstein, 2008). This unconditional and continuous quality means that family support provides what attachment theorists would call a “secure base” (Bowlby, 1969): a relational foundation from which individuals can explore and engage with the world—including digital environments—without fear of abandonment or relational rupture. The empowerment mechanism described above is therefore expected to operate most powerfully through family support.

Peer (friend) support, by contrast, is embedded in relationships characterized by mutual surveillance, reciprocal expectations, and informal social-norm enforcement (Berkman et al., 2000). Friends serve as significant others whose opinions and evaluations carry normative weight; they are likely to notice and comment on perceived excessive media use, and they provide alternative social engagements—face-to-face interaction and shared activities—that directly compete with solo SFV consumption for time and attention. The social-monitoring function of peer relationships may therefore constrain rather than enable digital immersion, and the availability of face-to-face peer interaction may reduce the salience of SFV as a social substitute. Peer support is consequently expected to show the weakest, potentially non-significant, positive association with SFV engagement.

Community participation occupies an intermediate position. Participation in structured community activities—exercise groups, hobby clubs, community centers—carries a distinctive feature not present in dyadic relationships: an externally imposed temporal and spatial structure (Cornwell et al., 2008; Mendes de Leon et al., 2003). Fixed meeting times, physical attendance requirements, and scheduled programming naturally regulate the time available for alternative activities, including SFV use, without requiring the kind of interpersonal monitoring characteristic of peer relationships. This structural constraint moderates engagement without compromising the individual’s privacy or autonomy. Community participation thus represents a natural moderating force that maintains social connectedness while organically limiting immersion.

The theoretical analysis of source-specific mechanisms generates a clear prediction about the ordering of effects on SFV engagement—and, by extension, on the relative contribution of the engagement pathway to downstream life satisfaction. Family support, operating via unconditional relational security, is expected to show the strongest association with SFV engagement; community participation, operating via partial social satiation bounded by structural constraint, is expected to show an intermediate effect; and peer support, whose empowerment potential is suppressed by monitoring and displacement, is expected to show the weakest effect. This ordering constitutes the study’s primary source-differentiation hypothesis:

**H4:**  *The three social support sources will produce differential effects on SFV engagement, with a-path coefficients ordered as: family support > community participation > peer support, reflecting the empowerment, structural constraint, and monitoring/displacement mechanisms, respectively. Downstream effects on life satisfaction will mirror this ordering in total effect magnitude.*

### 1.5. The Present Study

Taken together, the preceding theoretical analysis identifies a critical gap in the existing literature: while the aggregate effects of social support on digital behavior and well-being among older adults have received increasing attention, the mechanisms by which different types of support shape these relationships remain poorly understood. Most studies treat social support as a single variable, and most theoretical accounts offer predictions at a level of generality that obscures the meaningful heterogeneity across support sources.

The present study addresses this gap by employing a chained mediation framework to examine three parallel pathways linking distinct social support sources (family, peer, community) to life satisfaction via SFV engagement and social connectedness, in a sample of 385 Chinese older adults aged 60 and above. By disaggregating social support by source and testing differential effects within a unified model, we seek to determine not merely whether social support matters for older adults’ digital lives, but which kind of support, through what mechanism, and with what consequences for well-being. In doing so, this study contributes a source-differentiated theoretical account of social empowerment. It offers granular evidence to inform the design of support systems and digital well-being policies in aging societies.

Taken together, these theoretical perspectives are not competing accounts but complementary lenses that operate at different levels of analysis. Social empowerment theory provides the overarching framework: it identifies the direction of the support–engagement relationship and reframes immersive SFV use as an expression of relational security rather than as a sign of deprivation. Self-Determination Theory and attachment theory provide the psychological mechanisms through which this empowerment operates—need satisfaction and secure-base exploration, respectively—explaining why fulfillment of relatedness enables autonomous leisure. Uses and Gratifications Theory contextualizes the purposive, agentic quality of older adults’ media selection. At the same time, Compensatory Internet Use Theory anchors the study’s contribution by specifying the alternative (deficit-driven) pathway that the empowerment account challenges. The displacement hypothesis completes the framework by accounting for the potential costs of sustained engagement. This integrated framework, rather than any single theory in isolation, guides the hypotheses and interpretations that follow.

**Figure 1:**
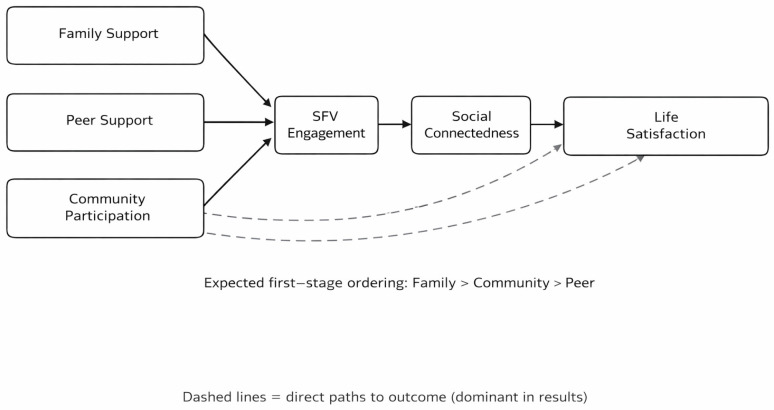
Source-Differentiated Conceptual Framework of the Present Study. Family support, peer support, and community participation were modeled as parallel predictors, with both direct and indirect paths to life satisfaction. SFV engagement and social connectedness were specified as sequential mediators. Dashed lines indicate direct effects of each support source on life satisfaction.

Beyond serving as interpretive resources, the theories mobilized in this study directly shaped the research design. First, the integration of social empowerment theory, Self-Determination Theory, and attachment theory indicated that social support should not be modeled as a single aggregate resource. Instead, family, peer, and community support needed to be treated as analytically distinct predictors because they imply different relational mechanisms. Second, the combination of empowerment logic and the displacement hypothesis suggested a sequential process in which the support source would shape SFV engagement, which could then influence social connectedness and ultimately life satisfaction. This logic justified the use of a parallel serial mediation design rather than an aggregate-support or single-path model. Third, these theories informed the development of source-specific hypotheses: family support was expected to show the strongest positive association with SFV engagement, community participation an intermediate association, and peer support the weakest association due to monitoring and displacement. In this sense, theory in the present study does not merely explain the findings after the fact; it organizes variable selection, model structure, and hypothesis development from the outset. Figure 1 presents the conceptual framework of the present study.

## 2. Methods

### 2.1. Participants and Procedure

Participants were community-dwelling adults aged 60 years and above residing in mainland China. Data were collected between July and August 2025 via Credamo (www.credamo.com), a professional online research panel widely used in Chinese social science research that maintains participant pools with verified demographic profiles. Credamo employs quota-based sampling procedures and applies quality-control measures, including attention checks, response-time monitoring, and duplicate-IP filtering. The sample’s demographic profile broadly resembled national statistics for this age cohort (CNNIC, 2025), though the non-probabilistic sampling method precludes claims of strict population representativeness.

Eligibility criteria were: (1) age 60 or above, (2) current user of at least one short-form video platform (e.g., Douyin, Kuaishou), and (3) provision of written informed consent before survey commencement. Participants who reported no SFV use were excluded at screening to ensure construct validity of the engagement measures. A total of 410 responses were initially received. To ensure data quality, responses were screened manually using two criteria: completion time falling below the 10th percentile of the sample distribution, and straight-line response patterns (i.e., identical responses across all items). Following the removal of 25 invalid responses on these grounds, the final analytic sample comprised *N* = 385 participants.

Sample characteristics were as follows: mean age = 70.6 years (*SD* = 7.3, range = 60–91); 49.2% male; mean monthly income = 2.93 thousand CNY (*SD* = 1.56); mean self-rated health = 3.05 (*SD* = 1.04) on a five-point scale (1 = very poor, 5 = very good). A power analysis conducted a priori using G*Power 3.1 (Faul et al., 2007) indicated that a sample of 385 provided adequate statistical power (1 − β > 0.95) to detect small-to-medium effect sizes (f^2^ = 0.05) in a multiple regression framework with up to eight predictors (α = 0.05).

The present study constitutes the second wave of data collection within a broader research project. The first wave, reported in Cui et al. (2026), examined the effects of aggregate social support on SFV engagement and life satisfaction using an independently recruited sample collected via the same platform during the same period. The present study extends that work by disaggregating social support into three theoretically distinct sources—family, peer, and community—and testing source-differentiated mediation pathways across a new, independent sample (Wave 1: *N* = 384; Wave 2: *N* = 385). The substantially different descriptive profiles across the two waves (e.g., life satisfaction: Wave 1 *M* = 2.89 vs. Wave 2 *M* = 3.38; social connectedness: Wave 1 *M* = 3.58 vs. Wave 2 *M* = 3.24) confirm the independence of the two samples.

### 2.2. Measures

All measures employed five-point Likert-type response scales (1 = strongly disagree to 5 = strongly agree) unless otherwise noted. Items were presented in Simplified Chinese; all instruments used were validated Chinese-language versions with established psychometric properties in older adult populations. Descriptive statistics and reliability estimates for all measures are presented in Table 1.

#### 2.2.1. Social Support Sources

Social support was assessed using the Social Support Rating Scale (SSRS; Xiao, 1994), a widely validated instrument extensively used in Chinese aging research. Rather than using the SSRS total score, we operationalized three theoretically distinct support sources as separate predictors, consistent with our source-differentiated theoretical framework and with the item-level structure of the SSRS (Xiao, 1994).

Peer support was indexed by a single SSRS item assessing the number of close friends from whom the respondent could receive support and help: “How many close friends do you have who can provide you with support and help?” (*M* = 3.25, *SD* = 1.29). We acknowledge the psychometric limitations of single-item measurement; its implications are discussed in the Limitations section.

Family support was operationalized as the mean of two SSRS items: one assessing the level of support and care received from family members (“I receive considerable support and care from my family members”), and one assessing the respondent’s tendency to confide in others when troubled (“When you encounter difficulties, do you confide in others?”) (*M* = 3.27, *SD* = 0.97; inter-item *r* = 0.48, *p* < 0.001).

Community participation was operationalized as the mean of two SSRS items: one assessing the quality of relationships with neighbors, and one assessing frequency of participation in organized community group activities such as party organizations, religious organizations, community activity centers, or senior universities (*M* = 3.27, *SD* = 0.99; inter-item *r* = 0.51, *p* < 0.001).

#### 2.2.2. Short-Form Video Engagement

SFV engagement was assessed using the Bergen Social Media Addiction Scale (BSMAS; Andreassen et al., 2017), adapted for short-form video use among Chinese older adults. We wish to be explicit about the construct being measured. In the present study, the adapted BSMAS operationalizes SFV engagement intensity—the degree of salience, habitual involvement, and behavioral investment in short-form video use—rather than clinical addiction in a diagnostic sense. This use is consistent with a growing literature that has adapted addiction-framework scales to capture high-intensity engagement in non-clinical populations, recognizing that the behavioral components indexed by such scales are continuous dimensions of engagement that extend well beyond pathological thresholds (Billieux et al., 2015). The core argument of this study—that SFV engagement among well-supported older adults reflects autonomous leisure rather than compensatory coping—is a claim about the psychological context of engagement, not a claim that high scores are benign regardless of context. Throughout this paper, we use “SFV engagement” rather than “addiction” in recognition of this distinction. The adaptation followed a minimal-modification approach (Beaton et al., 2000); therefore, all six original items were retained in their entirety, with two targeted changes only. First, references to “social media” in each item were replaced with “short-form video” (短视频) to align the construct with the specific platform behavior under investigation, consistent with prior adaptations in the Chinese digital media literature (Cui et al., 2026). Second, one item (Item 6: “You have used short-form video so much that it has had a negative impact on your life”) was supplemented with a parenthetical contextual example (e.g., “such as forgetting food on the stove”) to enhance comprehensibility for older adult respondents, without altering the item’s substantive meaning. No items were deleted, reordered, or otherwise reworded. The adapted items were reviewed for face validity by two researchers with expertise in aging and digital media prior to data collection.

The scale comprises six items reflecting the core behavioral addiction components: salience (C1), tolerance (C2), mood modification (C3), relapse (C4), withdrawal (C5), and conflict (C6). A sample item reads: “You spend a lot of time thinking about short-form video content, or planning what to watch next.” Total scores were computed by summing all six items (range: 6–30). The scale demonstrated good internal consistency in the present sample (Cronbach’s α = 0.855, *M* = 3.26, *SD* = 0.85). We note that no confirmatory factor analysis (CFA) was conducted on the adapted scale in the current sample; structural validity rests on the minimal nature of the adaptation and the strong internal consistency observed. The absence of CFA-based validation is acknowledged as a limitation. Higher scores indicate greater engagement intensity. Throughout this paper, we use the term “SFV engagement” rather than “addiction” when referring to scale scores, recognizing that scores at the mean level (as observed here) reflect habitual engagement rather than clinically defined addiction (Billieux et al., 2015).

#### 2.2.3. Social Connectedness

Social connectedness was measured using an eight-item abbreviated version of the UCLA Loneliness Scale (Russell, 1996). Items assess the subjective experience of social isolation and relational quality (e.g., “You feel that you lack companionship”; “You feel left out”). Consistent with prior research examining social connectedness as a positive psychological resource, items were reverse-scored so that higher values reflect greater perceived social connectedness rather than loneliness. The scale demonstrated acceptable internal consistency (α = 0.700, *M* = 3.24, *SD* = 0.57).

#### 2.2.4. Life Satisfaction

Life satisfaction was assessed using the Satisfaction with Life Scale (SWLS; Diener et al., 1985), a five-item measure widely used in aging research. Items include statements such as “In most ways my life is close to my ideal” and “I am satisfied with my life.” Total scores were computed by summing all five items (range: 5–25). The scale demonstrated good internal consistency in the present sample (α = 0.822, *M* = 3.38, *SD* = 0.81).

#### 2.2.5. Control Variables

Based on prior literature identifying demographic correlates of social media use and well-being in older adults (Nebenzahl-Elitzur et al., 2026; R. Zhang et al., 2024), four variables were included as covariates: age (continuous, in years), sex (binary: 0 = female, 1 = male), monthly income (in thousands of CNY, treated as continuous), and self-rated health (five-point scale: 1 = very poor to 5 = very good). All covariates were entered simultaneously at each step of the mediation model.

### 2.3. Analytic Strategy

Hypotheses were tested using a chained (serial) mediation framework with three parallel pathways estimated simultaneously. The model specified social support source (X: peer support, family support, and community participation, entered as three separate parallel predictors) as the independent variables, SFV engagement (M1) and social connectedness (M2) as sequential mediators, and life satisfaction (Y) as the outcome, with age, sex, income, and self-rated health as covariates at each step. This structure yielded three parallel chained mediation paths of the form: X → M1 → M2 → Y.

All variables were standardized prior to analysis (mean = 0, *SD* = 1) to facilitate comparison of path coefficients across predictors. Standardized regression coefficients (β) are reported throughout. Multicollinearity was assessed using variance inflation factors (VIF); all VIFs were below 2.5, confirming no problematic collinearity among predictors.

The significance of indirect (mediated) effects was evaluated using bias-corrected bootstrapped confidence intervals based on 5000 resampling iterations (Preacher & Hayes, 2008; Hayes, 2018). Indirect effects whose 95% confidence intervals excluded zero were interpreted as statistically significant. This nonparametric approach is preferred over the Sobel test because it makes no assumption of normality in the sampling distribution of the product of path coefficients and maintains adequate power with moderate sample sizes (MacKinnon et al., 2004). Analyses were conducted in SPSS 26.0 using the PROCESS macro (v4.2; Hayes, 2018), Model 6 (chained mediation).

### 2.4. Ethics Statement

The study was approved by the Biomedical Research Ethics Committee of Nanjing Normal University (Approval No. NNU202506056). All participants were fully informed of the study’s purpose, procedures, voluntary nature, and the confidentiality of their responses prior to participation. Written informed consent was obtained from all participants before survey commencement. No deception was employed. Data were anonymized at the point of collection and stored securely in accordance with institutional data governance protocols. The raw dataset, in CSV format with full variable documentation, is available upon reasonable request from qualified researchers, consistent with open science practices and institutional policy.

## 3. Results

### 3.1. Preliminary Analyses: Descriptive Statistics and Zero-Order Correlations

Descriptive statistics and zero-order correlations among all study variables are presented in Table 2. The three social support sources yielded comparable mean scores (peer support: *M* = 3.25, *SD* = 1.29; family support: *M* = 3.27, *SD* = 0.97; community participation: *M* = 3.27, *SD* = 0.99), indicating that the sample, on average, reported moderate and broadly balanced levels of support across sources. SFV engagement (*M* = 3.26, *SD* = 0.85), social connectedness (*M* = 3.24, *SD* = 0.57), and life satisfaction (*M* = 3.38, *SD* = 0.81) were similarly centered around the midpoint of their respective scales.

Several noteworthy patterns emerged in the correlation matrix. First, SFV engagement was strongly and positively correlated with life satisfaction (*r* = 0.409, *p* < 0.001), a pattern that superficially conflicts with pathologizing accounts of digital media use and is consistent with the “social empowerment” phenomenon described in the introduction. Second, the three support sources showed differentiated inter-correlations: family support and community participation were highly correlated (*r* = 0.569, *p* < 0.001), suggesting a “concentrated support” structure in which formal and informal community-level ties tend to co-occur; peer support was more weakly correlated with both family support (*r* = 0.314, *p* < 0.001) and community participation (*r* = 0.467, *p* < 0.001), indicating relative independence of the friendship network. Third, and theoretically significant, family support showed no significant correlation with social connectedness (*r* = −0.018, *ns*), whereas both peer support (*r* = −0.125, *p* < 0.05) and community participation (*r* = −0.127, *p* < 0.05) were associated with lower loneliness (i.e., higher connectedness). This divergence foreshadows that family support operates through a mechanism distinct from loneliness reduction—consistent with the empowerment account—while peer and community support more directly sustain social connectedness. All variables were within acceptable ranges for skewness and kurtosis (|skew| < 2.0, |kurtosis| < 7.0), satisfying assumptions for regression-based analysis.

### 3.2. Chained Mediation: Three Parallel Pathways

The chained mediation model (X → M1 → M2 → Y) was estimated simultaneously for three parallel support sources, with age, sex, income, and self-rated health entered as covariates at each step. Standardized path coefficients are reported. VIF values across all equations ranged from 1.08 to 2.31, indicating no problematic multicollinearity. Full path coefficients for the three parallel models are presented in Table 3.

#### 3.2.1. Pathway 1: Peer Support → SFV Engagement → Social Connectedness → Life Satisfaction

The path from peer support to SFV engagement (path *a*) was positive but non-significant (β = 0.023, *ns*), indicating that greater peer support did not meaningfully predict higher levels of SFV engagement. This result is consistent with the hypothesized monitoring-and-displacement mechanism: peer relationships provide alternative social outlets and informal normative regulation that jointly suppress the empowerment of immersive media use. SFV engagement negatively predicted social connectedness (path *d*_21_: β = −0.099, *p* < 0.05), and social connectedness positively predicted life satisfaction (path *b*_2_: β = 0.269, *p* < 0.001). The total effect of peer support on life satisfaction was significant (β = 0.164, *p* < 0.01), indicating a direct protective association. However, the chained indirect effect (path *a* × *d*_21_ × *b*_2_) was negligible and non-significant (β = −0.0006, 95% CI [−0.0047, 0.0025]), as the non-significant *a* path precluded meaningful mediated transmission.

#### 3.2.2. Pathway 2: Family Support → SFV Engagement → Social Connectedness → Life Satisfaction

Family support demonstrated the strongest association with SFV engagement of the three sources, with a significant positive path (β = 0.166, *p* < 0.01). This result directly supports H1a and the social empowerment account: older adults with more robust family relationships engage more deeply with SFV, not less—a pattern consistent with secure relational attachment enabling autonomous leisure behavior. The subsequent paths showed that SFV engagement negatively predicted social connectedness (β = −0.091, *ns* at conventional threshold), and social connectedness significantly predicted life satisfaction (β = 0.262, *p* < 0.001). The total effect of family support on life satisfaction was the largest among the three sources (β = 0.321, *p* < 0.001), underscoring the central role of family relationships in Chinese older adults’ subjective well-being. The chained indirect effect, however, was small and its 95% CI marginally included zero (β = −0.0040, 95% CI [−0.0107, 0.0008]), indicating that the mediation chain—while directionally consistent—did not reach conventional significance thresholds. The dominant mechanism through which family support operates is therefore direct rather than mediated through the engagement–connectedness chain.

#### 3.2.3. Pathway 3: Community Participation → SFV Engagement → Social Connectedness → Life Satisfaction

Community participation showed a moderate, significant positive association with SFV engagement (β = 0.115, *p* < 0.05), falling between the non-significant peer support pathway and the stronger family support pathway. This pattern is consistent with the structural constraint account: community activities provide enough social satiation to support some degree of empowered digital leisure, while their temporal and spatial structure organically limits the intensity of immersion. The path from SFV engagement to social connectedness was negative (β = −0.086), and the path from social connectedness to life satisfaction was positive (β = 0.282, *p* < 0.001), consistent with H2 and H3. The total effect of community participation on life satisfaction was substantial (β = 0.271, *p* < 0.001). The chained indirect effect was non-significant (β = −0.0028, 95% CI [−0.0089, 0.0022]).

### 3.3. Summary of Hypothesis Tests

The pattern of results provides partial support for the study’s hypotheses. H1a was supported (family support: β = 0.166, *p* < 0.01) and H1b was supported (community participation: β = 0.115, *p* < 0.05), while H1c was also confirmed—peer support showed negligible association with engagement (β = 0.023, *ns*), consistent with the monitoring and displacement prediction. H2 (SFV engagement negatively predicts social connectedness) received directional support across all three pathways, with the peer support pathway reaching significance (β = −0.099, *p* < 0.05); the effect was consistent but sub-threshold in the family and community pathways. H3 (social connectedness positively predicts life satisfaction) was robustly supported across all three pathways (β = 0.262–0.282, all *p* < 0.001). H4 (differential indirect effects by support source) was partially supported as the *a* paths showed the predicted ordering (family > community > peer), with a sevenfold difference in magnitude between the strongest (family: β = 0.166) and weakest (peer: β = 0.023) pathways. At the same time, the downstream portions of the mediation chain (*d*_21_ and *b*_2_) were highly consistent across sources. Notably, none of the three chained indirect effects reached significance, indicating that the support-to-satisfaction relationship is primarily direct rather than operating through the engagement–connectedness mediation chain.

## 4. Discussion

This study investigated whether different sources of social support—family, peers, and community—differentially affect short-form video engagement and life satisfaction among Chinese older adults, using a chained mediation framework. Three findings stand out. First, support source, not aggregate support quantity, is the decisive factor. The three sources predicted SFV engagement with markedly different strengths, with the a-path coefficients differing by a factor of seven between the strongest (family: β = 0.166) and weakest (peer: β = 0.023) predictors. Second, this source-level heterogeneity does not reflect different downstream psychological mechanisms—the engagement-to-connectedness and connectedness-to-satisfaction segments of the chain were nearly identical across all three pathways. Third, the dominant pathway from social support to life satisfaction is direct rather than mediated as none of the three chained indirect effects reached statistical significance, indicating that social support primarily acts as a direct psychological resource rather than through the engagement–loneliness chain. We discuss each finding in turn, situate it within the theoretical literature, and draw out its implications.

### 4.1. Family Support as the Primary Empowerment Resource

The most theoretically consequential finding is that family support most strongly and significantly predicted SFV engagement (β = 0.166, *p* < 0.01), and it also showed the strongest total association with life satisfaction (β = 0.321, *p* < 0.001). This pattern directly inverts the prediction of Compensatory Internet Use Theory, which would anticipate that strong offline support reduces the motivation for digital immersion. Instead, it aligns squarely with the social empowerment account.

The empowerment mechanism can be understood through the lens of attachment theory and Self-Determination Theory. Bowlby (1969) argued that secure attachment relationships function as a “haven” that reduces anxiety and a “secure base” from which individuals can explore their environment with confidence. Deci and Ryan (2000) extended this logic to motivational regulation, proposing that the satisfaction of relatedness needs creates the psychological conditions for autonomous—as opposed to controlled or compulsive—engagement with activities. When older adults are embedded in secure, unconditional family relationships, the need for relatedness is durably met in offline contexts. SFV engagement under these conditions carries a different psychological signature than compensatory use as it is not driven by loneliness or relational deprivation, but represents a freely chosen leisure activity that the individual can adopt, sustain, or abandon without existential anxiety. In this sense, family support does not merely permit digital engagement—it authorizes it as a legitimate expression of personal autonomy.

This interpretation has particular resonance in the Chinese cultural context. Despite the rapid erosion of multi-generational co-residence patterns accompanying urbanization, family relationships remain the primary locus of emotional security and social identity in Chinese society (Silverstein et al., 2006; Cong & Silverstein, 2008). The Confucian relational framework positions family belonging not merely as one social resource among others, but as the foundational condition of psychological selfhood. For Chinese older adults, a strong family support network may therefore provide a uniquely powerful base of relational security—one whose empowerment effects on autonomous behavior may be stronger than those observed in more individualist cultural contexts. Future cross-cultural research should test whether the family-support empowerment effect generalizes beyond Chinese samples.

Importantly, family support showed no significant zero-order correlation with social connectedness (*r* = −0.018, *ns*), suggesting its influence on well-being operates via a direct psychological pathway—heightened sense of security, self-worth, and relational belonging—rather than through reductions in loneliness. This profile distinguishes family support from the other two sources and underscores why aggregate social support measures, which collapse this distinction, systematically obscure a theoretically important source of heterogeneity.

### 4.2. Peer Support: The Monitoring-And-Displacement Paradox

Peer support’s non-significant association with SFV engagement (β = 0.023, *ns*) initially appears to disconfirm the empowerment hypothesis. We argue instead that it reflects a theoretically coherent suppression of the empowerment pathway by two competing mechanisms operating simultaneously.

One possible explanation, though not directly tested in the present study, involves social monitoring. Friendship networks may function, among other things, as informal regulators of normative behavior (Berkman et al., 2000; Umberson, 1987). Friends are uniquely positioned to notice and comment upon what they perceive as excessive or problematic media use—a form of benevolent surveillance that may operate through direct feedback (“You’re always on your phone”) as well as through the implicit awareness that one’s behavior is visible to and evaluated by significant others. This monitoring function may be more pronounced in peer relationships than in family relationships, where the unconditional nature of the bond provides more latitude for idiosyncratic behavior, or in community settings, where the relationship is less intimate and continuous.

A second possible mechanism is activity displacement. Friends provide a direct, accessible alternative to solo media consumption, as face-to-face interactions, shared outings, and joint activities compete with SFV engagement for the same temporal and attentional resources (Kraut et al., 1998; Verduyn et al., 2015). For older adults with active peer networks, the marginal utility of SFV engagement as a social or entertainment resource may be reduced precisely because the social needs that SFV might otherwise partially fulfill are already being met through offline peer interaction.

These two mechanisms—monitoring and displacement—appear to cancel out the empowerment effect that would otherwise be expected from peer support’s contribution to relational security. The net result is a near-zero association between peer support and SFV engagement. This finding carries an ironic implication: the “protective” function of peer relationships vis-à-vis media use simultaneously constrains personal leisure autonomy. Friends’ concern about excessive use may be experienced not only as supportive regulation but also as an infringement on self-directed behavior—a tension between social control and personal freedom that has received little attention in the aging and digital media literature.

### 4.3. Community Participation: Structural Constraint as Optimal Balance

Community participation showed an intermediate, significant association with SFV engagement (β = 0.115, *p* < 0.05), falling between the strong empowerment effect of family support and the null effect of peer support. This intermediate position reflects a qualitatively distinct mechanism that we characterize as a structural constraint.

Unlike family support, community participation does not provide the continuous, unconditional relational security that most powerfully enables autonomous engagement. Unlike peer relationships, community participation does not involve dyadic monitoring or the provision of close personal alternatives to digital leisure. What community activities provide is a temporal and spatial architecture: fixed meeting times, physical attendance requirements, and scheduled programming. These structural features impose natural upper limits on the time available for SFV engagement without requiring interpersonal surveillance or normative pressure. The result is a moderation of immersion intensity that is experienced as externally given—a feature of one’s schedule rather than a response to social judgment—and that therefore preserves individual autonomy even as it constrains behavior.

From a policy perspective, this “optimal balance” quality of community participation is potentially its most important characteristic. Structured community activities simultaneously sustain social connectedness (both peer and community participation showed significant negative correlations with loneliness, unlike family support), limit potentially excessive media use, and protect individual autonomy. These properties make community-based programming a particularly attractive target for aging-friendly digital well-being interventions, as discussed further below.

### 4.4. The Direct Dominance of Social Support on Life Satisfaction

It should be noted at the outset that the proposed serial mediation model was not empirically supported: none of the three chained indirect effects were statistically significant. This is an important null finding that we take seriously and interpret directly, rather than explaining away. A finding that nonetheless cuts across all three pathways—and that requires careful theoretical interpretation—is the consistent non-significance of the chained indirect effects. Despite the significant associations observed along individual segments of the mediation chain, the product of the three path coefficients (*a* × *d*_21_ × *b*_2_) was negligible for all three support sources. This indicates that the support-to-satisfaction relationship is primarily direct rather than mediated through the SFV engagement–social connectedness chain.

This result can be interpreted in two ways. The first, methodological, interpretation acknowledges that three-step chained mediation imposes multiplicative attenuation: even when each constituent path is statistically significant, their product can be extremely small—and the present study may have been underpowered to detect such small indirect effects despite adequate power for the total effects (cf. Fritz & MacKinnon, 2007). The non-significance of the indirect effects, therefore, does not rule out mediation; it may simply reflect the limits of the current design for detecting third-order effects.

The second, substantive, interpretation takes the non-significance at face value as evidence that social support’s influence on well-being in later life is primarily a direct psychological resource effect (Cohen & Wills, 1985; Lakey & Cohen, 2000) rather than a behavioral mediation effect. On this view, what matters is not the behavioral chain (support enables media use, which displaces social connection, which undermines satisfaction) but the direct psychological experience of being supported—the sense of belonging, security, and mattering that social relationships provide, regardless of their downstream behavioral consequences. This interpretation is consistent with the absence of a significant correlation between family support and social connectedness: family support promotes life satisfaction not by reducing loneliness but by directly providing what Baumeister and Leary (1995) called the fundamental need to belong.

Both interpretations have merit and are not mutually exclusive. We encourage future research to employ designs with greater statistical power—larger samples, longitudinal measurement, and possibly multi-level modeling—to adjudicate between them.

### 4.5. Advances over Wave 1 and Cross-Wave Comparison

The present findings can be situated within the broader two-wave project of which this study forms the second phase. Cui et al. (2026), using an independent sample from the same research project, identified a significant chained indirect effect from aggregate social support to life satisfaction via SFV engagement and social connectedness, and interpreted the negative connectedness-to-satisfaction path as reflecting a state of “high-quality solitude.” The present study advances this work in three respects.

First, disaggregating social support by source reveals that the empowerment effect documented in Wave 1 is not uniformly distributed: it is driven primarily by family support (β = 0.166, *p* < 0.01), with community participation showing a weaker intermediate effect (β = 0.115, *p* < 0.05) and peer support showing no significant effect on engagement (β = 0.023, *ns*). The aggregate empowerment effect observed in Wave 1 thus masks substantial source-level heterogeneity. Second, the chained indirect effects, while directionally consistent with Wave 1, did not reach significance in Wave 2. This pattern suggests that the significant mediated pathway in Wave 1 may have been driven largely by the direct psychological resource effects of family support rather than by the behavioral engagement–connectedness chain per se. When support is disaggregated and entered simultaneously as three parallel predictors, the variance attributable to each individual indirect path is attenuated, consistent with the interpretation offered in Section 4.4.

Third, the direction of the connectedness-to-satisfaction path diverges across waves: negative in Wave 1 (β = −0.227) and positive in Wave 2 (β = 0.262–0.282). Two interpretations are offered. Statistically, Cui et al. (2026) acknowledged a suppression effect in their model, which may have distorted the sign of the connectedness path under aggregate-support conditions. The present source-differentiated model, by explicitly partitioning support variance, may provide a less confounded estimate of the connectedness–satisfaction relationship, recovering its theoretically expected positive direction. Substantively, the two samples differ in their life satisfaction profiles (Wave 1 *M* = 2.89 vs. Wave 2 *M* = 3.38), suggesting that Wave 1 participants may have been drawn from a segment of the older adult population for whom solitary immersion serves a more compensatory function—a possibility that warrants direct investigation in future longitudinal research. Taken together, the cross-wave comparison underscores that the relationship between SFV engagement and well-being is not uniform but is conditioned by both the source structure of offline support and sample-level variation in well-being baseline.

### 4.6. Reconceptualizing Older Adults’ Digital Engagement

Taken together, the present findings challenge the dominant pathologizing narrative of older adults’ digital media use. Aggregate SFV engagement was strongly and positively correlated with life satisfaction (*r* = 0.409), and the two support sources most strongly associated with well-being (family and community) were positively—not negatively—associated with SFV engagement. These patterns are difficult to reconcile with a framework that treats high SFV engagement as inherently problematic or as a symptom of social deprivation.

We propose instead a reconceptualization that distinguishes autonomous from compensatory SFV engagement, a distinction that maps onto the source of social support rather than the intensity of use. Older adults embedded in strong family relationships engage with SFV from a position of relational security; their immersion is self-endorsed, contextually appropriate, and compatible with—indeed, enabled by—a rich offline social life. This form of engagement is better characterized as “deep leisure” (Stebbins, 2007) than as behavioral addiction, even when engagement intensity is high. The BSMAS scores at the sample mean (*M* = 3.26) represent habitual engagement within a broader life context that the present data suggest is generally positive rather than impaired.

This reconceptualization has direct implications for policy and practice. Aging-service providers who advise older adults to simply “use their phones less” risk pathologizing behavior that, in context, reflects social confidence, leisure autonomy, and modernity. A more nuanced approach grounded in the present findings would involve three source-specific strategies. First, for family support, aging-service providers can actively reinforce family-centered digital practices—for example, facilitating intergenerational SFV sharing activities that simultaneously strengthen family bonds and build older adults’ digital competence, thereby leveraging the empowerment mechanism directly. Second, for peer networks, program coordinators should be attentive to the monitoring-and-displacement dynamic: rather than treating peer social activity as a straightforward protective factor, interventions should seek to balance peer-mediated social regulation with respect for individual leisure autonomy—for instance, through peer digital literacy groups that normalize SFV use rather than stigmatize it. Third, for community participation, the structural constraint mechanism identified here suggests that sustaining access to structured community activities—senior centers, exercise groups, hobby clubs—represents an effective leverage point, as such activities simultaneously maintain social connectedness and provide natural temporal boundaries on media use without requiring interpersonal surveillance. Across all three strategies, intervention resources should be targeted toward older adults with low social support across all sources—for whom compensatory digital engagement may be genuinely problematic—rather than applied as blanket use-reduction messaging to a population whose engagement is, in context, a positive indicator of well-being.

### 4.7. Implications for Research Design and Hypothesis Development

The present study also carries implications for research design and hypothesis development in studies of older adults’ digital well-being. Prior work has often relied on aggregate social support measures and generic linear associations between support, media use, and well-being. Our findings suggest that such designs may be theoretically under-specified because they assume equivalence across support sources. By showing that family, peer, and community support differ primarily at the first stage of the model, this study demonstrates the value of source-differentiated design and mechanism-specific hypothesis building.

More broadly, the study shows that integrating theories at different levels—motivational, relational, and behavioral—can guide the formulation of more discriminating research questions and more precise hypotheses than undifferentiated support models allow. Rather than asking whether social support, in general, predicts older adults’ short-form video engagement and life satisfaction, future research should ask which support source matters, through which mechanism, and at which stage of the process. This shift from aggregate to source-specific theorization may help move the field beyond overly broad models toward more analytically precise explanations of digital well-being in later life.

### 4.8. Limitations and Future Directions

Several limitations of the present study warrant acknowledgment. First, the cross-sectional design precludes causal inference. While the mediation framework implies a temporal ordering (support → engagement → connectedness → satisfaction), the direction of effects cannot be established from concurrent data, and all path coefficients should be interpreted as associations rather than causal effects. Longitudinal or experimental designs are needed to test whether changes in social support produce the predicted downstream effects on digital engagement and well-being.

Second, peer support was indexed by a single item (“How many close friends do you have who can provide you with support and help?”), which constrains measurement precision and precludes standard reliability estimation. Single-item measures are susceptible to random measurement error and may underrepresent the multidimensional nature of peer support, potentially attenuating observed associations with downstream outcomes. The non-significance of the peer support *a*-path—the most notable null finding in the present study—may therefore reflect measurement inadequacy as much as, or instead of, the theoretical monitoring-and-displacement mechanism we have proposed. This ambiguity limits the confidence with which the null peer support finding can be interpreted as substantive evidence for the monitoring-and-displacement account. Future research should operationalize peer support using validated multi-item scales (e.g., the friendship subscale of the MOS Social Support Survey (Sherbourne & Stewart, 1991) or adapted items from the Lubben Social Network Scale) to obtain more stable, psychometrically adequate estimates and to disentangle measurement and theoretical explanations for the observed null effect.

Third, the sample was recruited via an online research panel (Credamo), which necessarily selects for older adults who are already digitally active and comfortable completing online surveys. This introduces a systematic sampling bias, as older adults with limited digital literacy, low income, or restricted internet access—groups among whom compensatory dynamics may be most operative—are systematically underrepresented. The conclusions of this study, therefore, most reliably apply to digitally active older adults and should not be extended uncritically to the broader older adult population. We note that online platform recruitment is common in this literature (e.g., Cui et al., 2026; R. Zhang et al., 2024), and the present findings are consistent with prior work using comparable sampling methods; nonetheless, probability-based sampling designs are needed to test the generalizability of the source-differentiated empowerment framework to less digitally integrated subgroups.

Fourth, the present study focused exclusively on Chinese older adults, and the cultural specificity of the findings—particularly the unusually strong empowerment effect of family support—remains to be assessed. Cross-national replication is needed to determine the extent to which findings reflect universal mechanisms versus culturally particular configurations of relational support.

Finally, the present model did not assess the content or quality of SFV engagement—whether older adults primarily consume entertainment, informational, or socially interactive content—or distinguish between active (content creation, commenting) and passive (scrolling, watching) modes of use. These distinctions may moderate the downstream effects on social connectedness and life satisfaction and represent a promising direction for future research.

Despite these limitations, the present study makes a substantive contribution by demonstrating that social support source heterogeneity—not total support quantity—is the key determinant of how offline social resources shape older adults’ digital engagement and well-being. The distinction between empowerment, monitoring, and structural constraint mechanisms provides a theoretically grounded vocabulary for understanding differential effects that aggregate social support measures have historically obscured.

## 5. Conclusions

The present study set out to address a deceptively simple question: which social ties matter for older adults’ digital engagement and well-being, and why? By disaggregating social support into three structurally distinct sources—family, peer, and community—and examining their differential effects within a unified chained mediation framework, we have shown that the answer depends critically on which ties are considered. Family support, operating through a relational security-based empowerment mechanism, most strongly predicts both SFV engagement and life satisfaction. Community participation, operating through structural constraint, achieves an intermediate balance between enabling digital leisure and sustaining social connectedness. Peer support, whose empowerment potential is suppressed by social monitoring and activity displacement, shows negligible effects on engagement while still contributing directly to well-being. Across all three pathways, social support exerts its influence on life satisfaction primarily through direct psychological mechanisms rather than through the behavioral chain of engagement and loneliness.

These findings carry a clear theoretical implication: social support is not a unitary resource, and treating it as one systematically obscures the mechanisms through which different relational contexts shape digital behavior in later life. The social empowerment phenomenon—whereby offline support enables rather than suppresses digital immersion—is not a property of “support” in general, but of family support specifically, and is best understood through the combined lens of Self-Determination Theory and attachment theory. Future research should adopt source-differentiated operationalizations as standard practice rather than relying on aggregate support indices.

For practitioners and policy makers, the core message is equally clear: older adults’ SFV engagement should not be treated as a uniform phenomenon to be reduced or managed. The appropriate response depends on the relational context. Where engagement is family-supported and autonomously motivated, it is better understood as a legitimate form of modern leisure than as a risk behavior. Where it occurs in the absence of adequate social support across all sources, more targeted intervention may be warranted. Building and sustaining community-based social infrastructure—structured activities that simultaneously provide connectedness and natural temporal boundaries on media use—represents the most actionable leverage point identified by this study. Ultimately, promoting well-being in an aging digital society requires moving beyond the question of how much older adults use short-form video and toward the more consequential question of the social conditions under which they do so.

## Figures and Tables

**Table 1:** Summary of measures, descriptive statistics, and reliability estimates (N = 385).

Variable	Scale	Items	M (*SD*)	Cronbach’s α
**Independent Variables: Social Support Sources (SSRS; Xiao, 1994)**				
Peer support	SSRS	1	3.25 (1.29)	—
Family support	SSRS	2	3.27 (0.97)	—
Community participation	SSRS	2	3.27 (0.99)	—
**Mediator 1 (M1): SFV Engagement (BSMAS; Andreassen et al., 2017)**				
Short-form video engagement	BSMAS	6	3.26 (0.85)	0.855
**Mediator 2 (M2): Social Connectedness (UCLA Loneliness Scale; Russell, 1996)**				
Social connectedness	UCLA	8	3.24 (0.57)	0.700
**Dependent Variable (Y): Life Satisfaction (SWLS; Diener et al., 1985)**				
Life satisfaction	SWLS	5	3.38 (0.81)	0.822
**Control Variables**				
Age (years)	—	—	70.6 (7.3)	—
Sex (% male)	—	—	49.2%	—
Monthly income (1000 CNY)	—	—	2.93 (1.56)	—
Self-rated health	—	—	3.05 (1.04)	—

*Note.* SSRS = Social Support Rating Scale; BSMAS = Bergen Social Media Addiction Scale; UCLA = UCLA Loneliness Scale; SWLS = Satisfaction with Life Scale. Social connectedness is reverse-scored such that higher values indicate stronger connectedness (lower loneliness). Cronbach’s α is not reported for single-item measures or demographic controls.

**Table 2:** Descriptive Statistics and Zero-Order Correlations Among Study Variables (N = 385).

Variable	*M*	*SD*	1	2	3	4	5	6
1. Peer support	3.25	1.29	-					
2. Family support	3.27	0.97	0.314 **	-				
3. Community participation	3.27	0.99	0.467 **	0.569 **	-			
4. SFV engagement	3.26	0.85	0.025	0.141 **	0.118 *	-		
5. Social connectedness	3.24	0.57	−0.125 *	−0.018	−0.127 *	−0.099 *	-	
6. Life satisfaction	3.38	0.81	0.164 **	0.321 **	0.271 **	0.409 **	0.269 **	-

*Note.* * *p* < 0.05. ** *p* < 0.01. Social connectedness is scored so that higher values reflect greater connectedness (lower loneliness).

**Table 3:** Standardized Path Coefficients for Three Parallel Chained Mediation Models (N = 385).

Pathway	a (X → M1)	*d*_21_ (M1 → M2)	*b*_2_ (M2 → Y)	Total Effect	Indirect Effect	95% CI
Peer support	0.023	−0.099 *	0.269 ***	0.164 **	−0.0006	[−0.0047, 0.0025]
Family support	0.166 **	−0.091	0.262 ***	0.321 ***	−0.0040	[−0.0107, 0.0008]
Community participation	0.115 *	−0.086	0.282 ***	0.271 ***	−0.0028	[−0.0089, 0.0022]

*Note.* Standardized β coefficients reported. All models controlled for age, sex, monthly income, and self-rated health. M1 = SFV engagement; M2 = social connectedness. Indirect effects estimated via bias-corrected bootstrapping with 5000 iterations. * *p* < 0.05. ** *p* < 0.01. *** *p* < 0.001.

## Data Availability

The data that support the findings of this study are available from the corresponding author upon reasonable request.
